# A New Endemic Focus of Chagas Disease in the Northern Region of Veraguas Province, Western Half Panama, Central America

**DOI:** 10.1371/journal.pone.0034657

**Published:** 2012-04-25

**Authors:** Azael Saldaña, Vanessa Pineda, Inri Martinez, Giovanna Santamaria, Ana Maria Santamaria, Aracelis Miranda, Jose E. Calzada

**Affiliations:** 1 Sección de Parasitología, Instituto Conmemorativo Gorgas de Estudios de la Salud (ICGES), Panama City, Panama; 2 Centro de Investigación y Diagnóstico de Enfermedades Parasitarias (CIDEP), Facultad de Medicina, Universidad de Panama, Panama City, Panama; 3 Centro de Salud de Santa Fe, Ministerio de Salud (MINSA), Santiago, Veraguas, Panama; 4 Hospital Dr. Luis Fabrega, Ministerio de Salud de Panamá (MINSA), Santiago, Veraguas, Panama; Weill Cornell Medical College, United States of America

## Abstract

**Background:**

Chagas disease was originally reported in Panama in 1931. Currently, the best knowledge of this zoonosis is restricted to studies done in historically endemic regions. However, little is known about the distribution and epidemiology of Chagas disease in other rural areas of the country.

**Methods and Findings:**

A cross-sectional descriptive study was carried out between May 2005 – July 2008 in four rural communities of the Santa Fe District, Veraguas Province. The study included an entomologic search to collect triatomines, bloodmeal type identification and infection rate with trypanosomes in collected vectors using a dot- blot and PCR analysis, genotyping of circulating *Trypanosoma cruzi* (mini-exon gene PCR analysis) and the detection of chagasic antibodies among inhabitants. The vector *Rhodnius pallescens* was more frequently found in La Culaca and El Pantano communities (788 specimens), where it was a sporadic household visitor. These triatomines presented darker coloration and larger sizescompared with typical specimens collected in Central Panama. *Triatoma dimidiata* was more common in Sabaneta de El Macho (162 specimens). In one small sub-region (El Macho), 60% of the houses were colonized by this vector. Of the examined *R. pallescens*, 54.7.0% (88/161) had fed on *Didelphis marsupialis*, and 24.6% (34/138) of *T. dimidiata* specimens collected inside houses were positive for human blood. *R. pallescens* presented an infection index with *T. cruzi* of 17.7% (24/136), with *T. rangeli* of 12.5% (17/136) and 50.7% (69/136) were mixed infections. In 117 *T. dimidiata* domestic specimens the infection index with *T. cruzi* was 21.4%. Lineage I of *T. cruzi* was confirmed circulating in these vectors. A *T. cruzi* infection seroprevalence of 2.3% (24/1,056) was found in this population.

**Conclusions:**

This is the first report of Chagas disease endemicity in Santa Fe District, and it should be considered a neglected public health problem in this area of Panama.

## Introduction

Chagas disease is a vector borne zoonosis caused by the kinetoplastid protozoan *Trypanosoma cruzi.* With nearly eight million people currently infected in 15 countries, it has been considered one of the most important parasitic diseases of the Americas [1], [2]. The systematic control of this parasitic infection has been coordinated in Latin America through a series of regional and multinational initiatives focused primarily on elimination of domestic triatomine vectors combined with health education and identification of transmission foci within a country [3], [4]. The epidemiological characterization of endemic areas including seroprevalence studies, ecology/biology of triatomine vectors, and parasite genotyping is an important prerequisite to establish effective control programs. This type of surveillance is ongoing in almost all endemic regions, even in countries where Chagas disease control has been successful [5], [6], [7], [8], [9]. Presently, the epidemiology of *T. cruzi* is well-known in most countries in Central America [10]. However, data from Panama concerning the epidemiological scenery of Chagas disease has been restricted to studies performed in traditional endemic communities in the central area of the isthmus [11], [12], [13], [14], [15]. Although Panama is a relatively small country (around 75,517.0 square kilometers), there are remote rural areas with potential ecological and socio-economic conditions adequate for Chagas disease transmission. Thus, concerning Chagas disease distribution and epidemiology, there are many regions in Panama that remain to be more carefully investigated. In this regard, little is known about the presence of triatomine vectors, trypanosome human infections and *T. cruzi* genetic characteristics found in the northern mountainous region of Veraguas Province in Western Panama.

During May-2005, some inhabitants from this rural area brought adults and nymphs of *Rhodnius pallescens* and *Triatoma dimidiata,* important vectors of Chagas disease in Panama [15], to the regional health center in the Santa Fe District in Western Panama. This incident alarmed health authorities of the possible existence of human *T. cruzi* infection cases in this region of the country. A cross sectional study was therefore carried out with the aim of confirming the presence and identity of triatomine vectors in houses, palm trees (*Attalea butyracea*) and other possible habitats, and to assess seropositivity for *T. cruzi* infections in the human population of Santa Fe District. These results provide valuable baseline information regarding a novel endemic focus of Chagas disease in the northern region of Veraguas province, and emphasize how Chagas disease epidemiology can vary over a small geographic area.

## Materials and Methods

### Ethics Statement

The study was approved by the National Review Board: Comite Nacional de Bioetica de la Investigacion, Instituto Conmemorativo Gorgas de Estudios de la Salud, Panama City, Panama (1424/CNBI/ICGES). All studied subjects provided written informed consent for all parts of the study.

### Area

**Figure 1:**
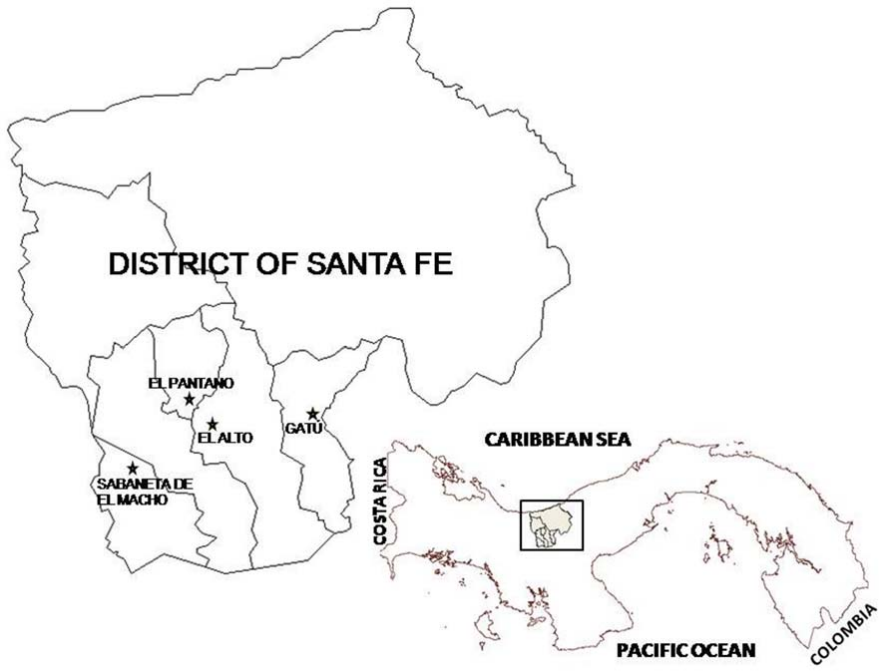
Map of the Santa Fe District, province of Veraguas, showing the geographic location of the surveyed communities.

The study was carried out between May 2005 and July 2008. The area is located in the northern region of Veraguas Province, Santa Fe district, in the western half of the Isthmus of Panama (Fig. 1). The main environmental characteristics of the evaluated areas are: a mountainous topography comprising many rural communities laying at different altitudes (400 to 1000 meters above the sea level); a mean temperature of 21°C; average annual rainfall of about 3,500 mm, and a marked seasonality with a dry season from December to March and a rainy season the rest of the year. The total population is approximately 15,000 inhabitants. Most of the district’s economy depends on subsistence agriculture. The surveyed specific regions were selected based on previous reports of triatomines found within the houses by their inhabitants. The evaluated communities were: El Pantano (8°33′ 0N, 81°5′ 0W, 604 masl) with approximately 183 houses and 495 inhabitants; La Culaca (8°31′ 0N, 81°2′ 60W, 698 masl) with approximately 61 houses and 188 inhabitants; Sabaneta del El Macho (8°29′ 29N, 81°7′ 40W, 805 masl) with approximately 149 houses and 488 inhabitants and Gatu (8°31′ 60N, 80°55′ 60W, 781 masl) with approximately 51 houses and 195 inhabitants.

### Triatomine Collection

Triatomine collection was conducted by community-based entomological surveillance, active searching of selected households by trained technical personnel, and dissection of peridomestic palms (*A. butyraceae*).

#### Community-based entomological surveillance

An entomologic search to collect triatomines was conducted with community participation. Briefly, plastic containers with perforated lids and filter paper in the form of an accordion inside, and with *R. pallescens* and *T. dimidiata* pictures adhered to its surface, were distributed among trained local inhabitants for the collection of bugs inside of approximately 444 houses distributed in the four evaluated regions. The captured bugs were forwarded by the house owner to the Health Center of Santa Fe and then to the Gorgas Institute Laboratory in Panama City for taxonomic identification and further evaluations.

#### Active collection by entomology specialists

Additionally, 30 selected houses from Sabaneta de El Macho were searched for triatomines by active collection prior to and after a first round of household deltametrin application by specialists from the Ministry of Health, Department of Vector Control. These houses were selected according to the following criteria: 1) a frequent household presence of *T. dimidiata* (based on resident reports), and 2) housing construction: walls made with a wooden framework packed with earth (“quincha”) and dirt floors.

#### Dissection of peridomestic palms

In Santa Fe District, as in others rural regions of the country, it is common practice to fell *A. butyracea* palm trees for construction of thatched roofs and for the production of a fermented beverage called “Palm Wine”. Triatomines were collected by direct search on crowns of these recently felled palms. All the investigated palms were located near houses (<100 mts). A total of 24 palms were evaluated, 15 palms from La Culaca, seven from El Pantano and two from Sabaneta de El Macho. The number, species, stages and morphological characteristics of collected triatomines were recorded.

### Host Feeding Source and Trypanosome Infection Analysis

The bloodmeal type and the presence/species of trypanosomes in the gut contents of those adult insects that arrived alive to the laboratory were identified by a dot-blot and PCR analysis as described [12], [14].

### Trypanosoma Cruzi Genotyping

The *T. cruzi* genotype lineages circulating in a subset of adult collected bugs was assessed according to previous reports [16], [17]. Briefly, DNA samples from fifty *T. cruzi* positive bugs (20 *R. pallescens* and 15 *T. dimidiata* collected on palm trees from La Culaca and 15 *T. dimidiata* found inside houses from Sabaneta de EL Macho) were analyzed using a multiplex-PCR assay based on amplification of the mini-exon gene, generating DNA bands of 300 or 350 bp for *T. cruzi* II (TCII) and *T. cruzi* I (TCI), respectively. The PCR amplified products were separated by electrophoresis in 2% agarose gels and stained with ethidium bromide.

### Blood Sampling

After obtaining written informed consent and with active participation of the Ministry of Health personnel, blood collection was performed on all persons older than two years old living in the four evaluated communities. Due to difficulties to transport/processing of blood samples in some of the evaluated communities (remote mountainous areas far from health centers) two standard procedures for blood collection were used: finger pricks blotted on filter paper using disposable lancets, or standard venipuncture.

### Serologic Analysis

To collect antibodies from filter papers, a 5 mm diameter punch was obtained from every sample and incubated overnight at 4°C in 200 µl of phosphate-buffered saline (PBS) using 96 well polystyrene plates. The filter paper eluates or sera samples were tested for anti-*T. cruzi* antibodies following procedures of two commercial assays (ELISA Chagatest, Wiener Laboratory, Argentina and Chagas Stat-Pak, Chembio Diagnostic Systems, Medgford, NY) and a western blot test described before [18]. Samples were considered positive when they showed reactivity in at least two of these tests.

### Data Analysis

Field data were collected by our research group in collaboration with medical personnel from the Health Center of Santa Fe and the Hospital Dr. Luis Fabrega in the city of Santiago. Field and laboratory data were recorded in notebooks and tables designed for this porpoise. Finally, tabulation, management and analysis of raw data were carried out using Microsoft Excel and Microsoft Access 2007 (Microsoft, Inc., United States).

## Results

### Triatomine Collection and Identification

Results of the entomological evaluation are depicted in Tables 1 and 2. Both *R. pallescens* and *T. dimidiata* were found in domestic habitats and in *A. butyracea* palm crowns. A total of 831 and 292 specimens of *R. pallescens* and *T. dimidiata* were collected, respectively. Other potential triatomine vectors, *Panstrongylus rufotuberculatus* (two specimens) and *Panstrongylus geniculatus* (one specimen), were rarely found inside houses. *R. pallescens* collected from this region presented an overall darker coloration and larger size (22–24 mm) compared with typical specimens collected in central Panama.

**Table 1:** Triatomine species captured in the houses/palm trees per communities in Santa Fe District.

	Community											
	La Culaca			El Pantano			Sabaneta de EL Macho			Gatu		
Triatomine	TCT	DCT (Ih/Eh)	WCT (Ip/Ep)	TCT	DCT (Ih/Eh)	WCT (Ip/Ep)	TCT	DCT (Ih/Eh)	WCT (Ip/Ep)	TCT	DCT (Ih/Eh)	WCT (Ip/Ep)
*R. pallescens*	689	15 (4/61)	674 (15/15)	99	6 (2/183)	93 (7/8)	43	– (–/149)	43 (2/2)	–	– (–/51)	–
*T. dimidiata*	100	1 (1/61)	99 (6/15)	30	– (–/183)	30 (4/8)	162	162 (20/149)	–(–/2)	-	– (–/51)	–

TCT = Total collected triatomines.

DCT (Ih/Eh) = Domestic collected triatomines (Infested houses/Evaluated houses).

WCT (Ip/Ep) = Wild collected triatomines (Infested palms/Evaluated palms).

**Table 2:** Triatomine feeding patterns and trypanosomes infection^1^.

	Evaluated triatomines	
Feeding Sources	*R. pallescens* 3(%)	*T. dimidiata* 4(%)
Opossum	88/161 (54.7)	3/138 (2.2)
Human	1/161 (0.6)	34/138 (24.6)
Bird	1/161 (0.6)	5/138 (3.6)
Dog	1/161 (0.6)	8/138 (5.8)
Mouse	5/161 (3.1)	3/138 (2.2)
Negative^2^	65/161 (40.4)	53/138 (38.4)
**Trypanosome infection**		
*T. cruzi* (+)	24/136 (17.7)	25/117 (21.4)
*T. rangeli* (+)	17/136 (12.5)	0/117 (0)
Mix infection	69/136 (50.7)	0/117 (0)
Negative	26/136 (19.1)	92/117 (78.6)

1 Results for those adult bugs that arrived alive to the laboratory.

2 Bugs that did not contain enough ingested blood for host identification or the blood of other no tested animals was present.

3 Bugs collected on palm trees from La Culaca and El Pantano regions.

4 Bugs collected inside houses from Sabaneta de El Macho.

This triatomine species was more frequently found in La Culaca and El Pantano communities (Table 1). In these areas the dwelling construction types were variable: 18/61 in La Culaca and 38/183 in El Pantano were constructed entirely with natural available materials in the region, such as bamboo pipes and palm leaves, and have dirt floors. However, there were also houses with plastered brick walls, cement floor, zinc-covered roof, and an electric supply.

The presence of *A. butyracea* palms in the backyards of the evaluated houses was common in these communities. Of these palms, 15/15 in La Culaca and 7/8 in El Pantano were infested with triatomines, with approximately 35 triatomines/palm. Direct observations confirm the presence of opossums, rats, bats and others synanthropic mammals in the peridomestic areas of these near communities. Interestingly, *R. pallescens* was a common visitor of some households in the La Culaca community and nymphal stages were occasionally encountered inside two of four infested houses. Despite these findings, constant and sustainable domestic colonies were not demonstrated in these houses during the study.

A different eco-epidemiological situation was found for *T. dimidiata.* This triatomine was most common in Sabaneta de El Macho (Table 1). Of 149 homes evaluated, 20 (12.0%) were colonized with *T. dimidiata.* The majority of these colonized houses (60%, 18/30) were located in near a mountain slope called El Macho. In this area, *T. dimidiata* was found in bedrooms (walls and beds), in walls crevices, and in accumulated objects from the infested houses. The average number of triatomines per house was 6 in El Macho area. Hygiene and building techniques were poor in these houses. Domestic animals (dogs, chickens, pigeons, etc) shared houses with the owners. Houses were constructed using a wooden framework packed with mud to form the walls. They had few windows and houses remained very dark even during day time. As no electricity is available, houses are commonly illuminated by kerosene lamps or candles at night. The inhabitants of this village are farmers and readily recognized adults and nymphs of *T. dimidiata.* Village inhabitants also mentioned they knew that *T. dimidiata* “eat” blood from human and domestic animals. However, they never had heard about Chagas disease before and were not aware of its transmission mode. Very few *A. butyracea* palm trees were found in Sabaneta de El Macho area; this probably explains the apparently low frequency of *R. pallescens* in this region of the Santa Fe District. *T. dimidiata* was also found in La Culaca and El Pantano communities (Table 1). However, most of these specimens (129/130, 99.2%) were collected in the palm tree crowns, and only one was collected inside a house from La Culaca.

### Host Feeding Source and Trypanosome Infection Analysis

It was found that 54.7% (88/161) of the examined *R. pallescens* had fed on common opossums (*Didelphis marsupialis*). These triatomines were collected on palm trees from La Culaca and El Pantano regions. The finding of positive triatomines for others feeding sources was scarce (Table 2). A significant number of *T. dimidiata* adult specimens collected inside houses were from Sabaneta de El Macho were positive for human blood, 34/138 (24.6%). Blood of domestic animals (chicken, dog and mouse) was also identified in the gut contents of 16 *T. dimidiata* (11.6%) found in the human domicile of Sabaneta de El Macho (Table 2). Only 2.2% (3/138) of these bugs were positive for opossum blood. Many of the evaluated triatomines (65 *R. pallescens* and 53 *T. dimidiata)* did not contain enough ingested blood for host identification or perhaps the blood of other animals that were not detectable by the dot-blot analysis were present. These results are referred as negative (Table 2).

In *R. pallescens* evaluated adults, the infection index with *T. cruzi* was 17.7% (24/136), and with *T. rangeli* 12.5% (17/136) for singly infected bugs. Around 50.7% (69/136) of these triatomines presented mixed infections with both trypanosomes (Table 2). In 117 *T. dimidiata* domestic specimens from Sabaneta de El Macho village, the infection index with *T. cruzi* was 21.4% (Table 1). The infection index with *T. cruzi* in 24 evaluated *T. dimidiata* bugs collected on palm trees from El Pantano and La Culaca communities was 75% (18/24).

### Trypanosoma Cruzi Genotyping

Fifty *T. cruzi* DNA samples isolated from infected *T. dimidiata* and *R. pallescens* collected inside houses and on palm trees were genotyped, all of them showed characteristic products (350 pb) of *T. cruzi* Lineage I using the mini-exon gene PCR analysis.

### Serologic Analysis

The combined information obtained from these four communities in Santa Fe District revealed a general *T. cruzi* infection seroprevalence of 2.3% (24/1,056) (Table 3). The highest seroprevalences were found in Sabaneta de El Macho (1.9%, 8/418,) and in El Pantano (3.6%, 12/328,). Only two positive cases were detected respectively on people evaluated from La Culaca (1.6%, 2/122) and Gatu (1.1%, 2/188) areas. Some positive cases were found in triatomine infested houses: La Culaca (1), El Pantano (2) and Sabaneta de El Macho (3). Most positives cases were detected in patients older than 50 years (45.8%, 11/24); however four infected young persons were also diagnosed (16.7%, 4/24). The seropositive patients were referred to the Santa Fe Health Center and the Dr. Luis Fabrega Hospital (Santiago City) for further evaluation, and in the cases of asymptomatic patients below 19 years old, for specific treatment for *T. cruzi* infection, following national guidelines for Chagas disease control.

**Table 3:** Seroprevalence of human *Trypanosoma cruzi* infection in the evaluated communities of Santa Fe district.

Community	Evaluated patients(% of total population)	Positive cases (%)	Positive cases found in infested houses (%)	Age of positive cases (years)		
				<19 (%)	19–50 (%)	>50 (%)
La Culaca	122 (64.9)	2 (1.6)	1 (50)	1 (50.0)	1 (50.0)	0 (0)
El Pantano	328 (66.3)	12 (3.6)	2 (16.7)	1 (8.3)	2 (16.7)	9 (75.0)
Sabaneta de El Macho	418 (85.7)	8 (1.9)	3 (37.5)	2 (25.0)	4 (50.0)	2 (25.0)
Gatu	188 (96.4)	2 (1.1)	0 (0)	0 (0)	2 (100.0)	0 (0)
Total	1,056 (77.3)	24 (2.3)	6 (25.0)	4 (16.7)	9 (37.5)	11 (45.8)

## Discussion

This study describes a new endemic area of Chagas disease in Panama, located in the mountainous and rural area of Santa Fe district. Over a four year’s period, two partially different epidemiological scenarios were evidenced in this region. *R. pallescens* and *T. dimidiata,* the two most important Chagas disease vectors in Panama [15], were collected during the entomological surveillance performed in the evaluated areas. Although *R. pallescens* was the predominant species, especially in La Culaca and El Pantano communities, our results also evidenced the existence of a far less common epidemiological situation reported in Panama where domiciliated *T. dimidiata* was the main vector found in Sabaneta de El Macho. These results emphasize that heterogeneity in Chagas disease epidemiologic scenarios can occur across a relatively small geographic area.

The predominance of *R. pallescens* in some sub-regions as compared to others may be related to the abundance of *A. butyracea* palm trees. In this sense, *A. butyracea* palms are common on the landscape of La Culaca and El Pantano, but scarce on the communities of Sabaneta de El Macho and Gatu. In most Chagas endemic areas of Panama, these palms are frequently infested with triatomine bugs, especially *R. pallescens* [12], [19]. The situation is similar in La Culaca and El Pantano communities, where more than 95% of the evaluated palms were infested with *R. pallescens*, around 767 specimens were collected from 22 dissected palms from these areas. As in other endemic regions [12], [20], these sylvatic triatomines presented high indices of infection with *T. cruzi* and/or *T. rangeli,* and have opossums as the main feeding blood source (Table 2). The opossums are the principal reservoir of Chagas disease in Panama [11].

The serological evaluation of 450 inhabitants from El Pantano and La Culaca shows that *T. cruzi* human transmission is occurring in this rural region of Panama (Table 3). Most endemic areas for Chagas disease in Panama have human seroprevalence lower than 5.0% (13,14,20). A comparable situation was found in these communities where the combined seroprevalence was 3.1%, including four cases of persons less than nineteen years old living in houses infested with triatomines, suggesting an active transmission. As in many others Panamanian endemic areas where *R. pallescens* is the main vector of Chagas disease [12], [13], [14], [20], the occurrence of *T. cruzi* and *T. rangeli* human infections in El Pantano and La Culaca counties is probably the result of flight dispersal of this triatomine from sylvatic zoonotic foci on near infested palm trees. Although *R. pallescens* nymphal stages were occasionally found inside these houses, as observed in La Culaca community, it is unlikely that this species can build up significant domestic populations in communities of Santa Fe district. Except for the pioneer work done by A.C. Pinking in 1968 [21], very few if any studies have considered some type of adaptation of *R. pallescens* to dwellings in rural areas of Panama.

*Triatoma dimidiata* was also frequently found on *A. butyracea* palm crowns from evaluated areas. Around 129 specimens of *T. dimidiata* were collected on these trees. This species is considered the second most important Chagas disease vector in Panama [15]. It has a broad distribution across the country, but generally at low density [15]. Sabaneta de El Macho is considered poorest area of the Santa Fe district, evidenced by the home construction: walls made with “quincha”, dirt floors, few windows and basic hygiene deficiencies. This environment has been described as ideal for the establishment of *T. dimidiata* colonies [22]. In fact, in 18% of the total evaluated houses (n = 144) houses in this region were colonized by *T. dimidiata*. Furthermore, *T. cruzi* infection index in these triatomines was 21.4% and 24.6% presented human blood in its intestine. The prevalence of antibodies against *T. cruzi* in this community was of 1.9% (8/418). Altogether, these findings clearly demonstrate that the inhabitants of this village live under a high risk for Chagas disease vectorial transmission. Thus, governmental implementation of a periodical surveillance and control programs is urgently needed in this area. These control strategies should integrate both the residual insecticide spraying and an improved management of domestic and peridomestic environments as recently suggested [22], [23].

Interestingly, no triatomine vectors were collected in the Gatu community. In this area the presence of *A. butyracea* palm was scarce and only two adult persons out of 188 evaluated presented antibodies against *T. cruzi.* Although these findings might suggest that Chagas disease is less common in Gatu area, additional studies are necessary to clarify the real status of this neglected parasitosis in this community.

The morphology of collected *T. dimidiata* specimens was apparently similar to those found in other Panamanian endemic regions [24]. In contrast, *R. pallescens* collected in Santa Fe district had an appreciable larger size and darker colour compared to specimens described from other Chagas disease endemic areas in Panama [24]. Other variations in colour patterns have been described for triatomines [25], [26], [27]. However, the epidemiological relevance of these observations needs additional investigations.

The molecular analysis of fifty *T. cruzi* isolates from infected *R. pallescens* and *T. dimidiata* collected both inside houses or on palm trees, revealed that the TCI is the predominant lineage. A similar situation have been described in two previous reports that analyzed *T. cruzi* strains isolated from chagasic patients and from *R. pallescens* collected in central Panama [17], [28]. However, it must be kept in mind that molecular characterization of parasites isolated from autochthonous patients is necessary before confirming that TCI is the main lineage associated with the human *T. cruzi* infections found in this region.

Based on our seroprevalence results, with a low number of samples, it seems very likely that an important number of Chagas disease cases from the Santa Fe District go undetected and that the few seropositive cases detected during this study may underestimate the true prevalence. Recently, a fatal case of chronic Chagas disease was reported at the regional Hospital “Dr. Luis Fabrega” in Santiago City in May of 2010. The patient came from El Pantano community, one of our evaluated areas, and presented a heart failure compatible with chronic Chagas disease and a positive serology for *T. cruzi.* Local physicians and other health personnel must be alert and trained to recognize signs and symptoms of Chagas disease, especially on patients from Santa Fe district.

In conclusion, this report demonstrates that Chagas disease is endemic in communities from Santa Fe District, and should be considered as a neglected public health problem in this rural region of Panama. Additionally, there is small-scale heterogeneity in the ecoepidemiology of Chagas disease vectors in this region, and this needs to be taken into account when designing future surveillance and disease control programs for Chagas disease in Panama.
